# A Shift from Efficiency to Adaptability: Recent Progress in Biomimetic Interactive Soft Robotics in Wet Environments

**DOI:** 10.1002/advs.202104347

**Published:** 2022-01-24

**Authors:** Jielun Fang, Yanfeng Zhuang, Kailang Liu, Zhuo Chen, Zhou Liu, Tiantian Kong, Jianhong Xu, Cheng Qi

**Affiliations:** ^1^ Department of Biomedical Engineering School of Medicine Shenzhen University Shenzhen Guangdong 518000 China; ^2^ College of Mechatronics and Control Engineering Shenzhen University Shenzhen 518000 China; ^3^ College of Chemistry and Environmental Engineering Shenzhen University Shenzhen Guangdong 518000 China; ^4^ The State Key Laboratory of Chemical Engineering Department of Chemical Engineering Tsinghua University Beijing 100084 China

**Keywords:** actuator, hydrogels, interactive, soft robotics, underwater

## Abstract

Research field of soft robotics develops exponentially since it opens up many imaginations, such as human‐interactive robot, wearable robots, and transformable robots in unpredictable environments. Wet environments such as sea and in vivo represent dynamic and unstructured environments that adaptive soft robots can reach their potentials. Recent progresses in soft hybridized robotics performing tasks underwater herald a diversity of interactive soft robotics in wet environments. Here, the development of soft robots in wet environments is reviewed. The authors recapitulate biomimetic inspirations, recent advances in soft matter materials, representative fabrication techniques, system integration, and exemplary functions for underwater soft robots. The authors consider the key challenges the field faces in engineering material, software, and hardware that can bring highly intelligent soft robots into real world.

## Graphical abstract

Recent progresses of soft robots performing tasks underwater herald a diversity of interactive soft robots in wet environments. Here, biomimetic inspirations, advances in soft matter materials, representative fabrication techniques, and exemplary functions are recapitualted for underwater soft robots. The authors consider key challenges in engineering material, software, and hardware that can highly intelligent soft robots into real world.

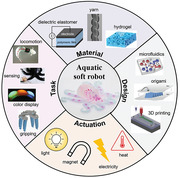

## 1 Introduction

Emerging paradigms like Internet of Things (IoT) and human–machine interfacing set off frontier technologies of soft robotics. Soft robots are robots made from elastomers, gels, and other easily deformable matters. Owing to its softness and compliance, a soft robot can serve as an intermediary between humans and machines. When combined with soft electronics and artificial intelligence (AI), soft robots and hybridized systems hold the promise for bridging human with hard robots and electronic computers for performing tasks and processing information.

Prior to future with neuro‐control interface, wearable computing and augmented body, soft robots can already provide unique advantages over conventional robots. Conventional robots are commonly rigid, heavy, and highly complex in design. They can exert large forces, and accomplish sophisticated tasks with high precision. However, they bend and straighten only around fixed points; their inflexibility makes them incapable of coping with dynamic, unstructured, or confined environments. Thus, they can be dangerous when dealing with adaptable humans, animals, and unpredictable situations. Inspired by boneless biological organisms, soft robots recapitulate the softness, compliance, and responsiveness of soft‐bodied creatures. They can stretch, twist, and squeeze into narrow spaces, they can transform into configurations upon environmental stimuli, and they can grip objects with soft touches, bringing inherent safety when interacting with human and animals. Soft robots with fundamentally different solutions from conventional hard robots are new classes of machines.

**Figure 1:**
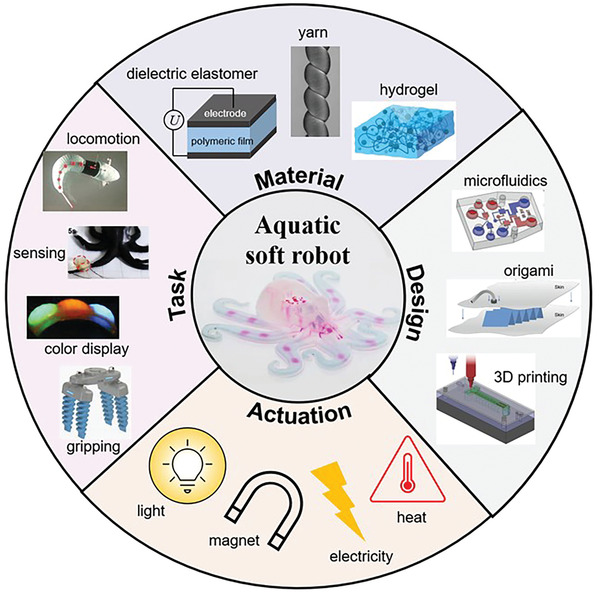
A schematic representation of contents discussed in this review.

The field of soft robotics has expanded rapidly, and its advances have been summarized in reviews with various focuses, namely, the actuation mechanisms of soft robots, novel hydrogels for constructing soft robots, and the next‐generation blueprints of drug delivery robots.^[^ ^1^, ^2^, ^3^, ^4^, ^5^, ^6^, ^7^, ^8^ ^]^ To unleash its full potentials, the capabilities of soft robots to intelligently interact with surrounding environments, specially, wet environments, are vital for their uses underwater and in vivo. This poses challenges, to name a few, integration, stability, durability, and environmental compatibility to be addressed. Recent progresses demonstrated soft hybridized robots performing simple tasks in wet/moist conditions, bringing us closer to interactive soft robots in wet environments. However, there exists inadequate state‐of‐art reviews focusing on progresses and future challenges of interactive soft robotics in wet/moist environments. The structure of this review follows the basic principles for developing biomimicry soft robots. Inspirations of aquatic and amphibious organisms are first discussed. Their functions are categorized, which are mimicked by aquatic soft robots. Suitable soft matter materials and representative fabrication techniques for aquatic soft robotics are overviewed. This is followed by detailed accounts of implemented functions of state‐of‐the‐art soft robots in wet environments, as well as exemplary demonstrations of these functions (**Figure** **1**). This review is closed by a brief discussion on unaddressed challenges and future directions.

## 2 Inspirations from Aquatic and Amphibious Organism

Nature, rich sources of inspirations, has iterated solutions that continuously motivate human engineering achievements. For instance, inspired by hard‐skeleton birds, scientists and engineers have worked out hard‐bodied robotic aircrafts. Naturally, when designing soft robots, soft‐bodied, and flexible creatures with high agility, such as octopuses, fishes, squids, starfish, and jellyfishes are sources of inspirations.

Most soft‐bodied organisms without skeletons are living in aquatic environments, since a mechanically stiff and heavy skeleton is not a necessity for surviving owing to the buoyancy of water.^[^ ^9^ ^]^ As a result, these marine organisms have entirely different strategies for locomotion and manipulations from those of hard‐bodied animals. For instance, caterpillars escape danger by snapping into a circle and wheel away.^[^ ^10^ ^]^ Thus, designing and fabricating soft robots can potentially provide new capabilities that hard robots cannot provide.

**Figure 2:**
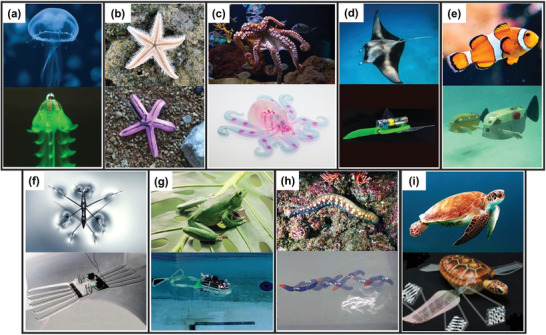
Representative images of living creatures and their robotic counterparts: a) jellyfish. Top photo by Thomas Millot on Unsplash, bottom reproduced with permission.^[^ ^347^ ^]^ Copyright 2019, Springer Nature. b) starfish. Top photo by shahd h on Unsplash, bottom reproduced with permission.^[^ ^19^ ^]^ Copyright 2014, Springer Nature. c) octopus. Top photo by Serena Repice Lentini on Unsplash, bottom reproduced with permission.^[^ ^20^ ^]^ Copyright 2016, Springer Nature. d) Manta ray. Top photo by Naushad Mohamed on Unsplash, bottom reproduced with permission.^[^ ^21^ ^]^ Copyright 2017, American Association for the Advancement of Science. e) fish. Top photo by Rachel Hisko on Unsplash, bottom reproduced with permission.^[^ ^348^ ^]^ Copyright 2017, IOP Publishing. f) water‐strider. Top photo by Tanguy Sauvin on Unsplash, bottom reproduced with permission.^[^ ^349^ ^]^ Copyright 2011, American Chemical Society. g) frog. Top photo by Joel Henry on Unsplash, bottom reproduced with permission.^[^ ^350^ ^]^ Copyright 2020, IOP Publishing. h) eel. Top photo by Francisco Jesús Navarro Hernández on Unsplash, bottom reproduced with permission.^[^ ^351^ ^]^ Copyright 2021, IOP Publishing. i) sea turtle. Photo by Wexor Tmg on Unsplash, Reproduced with permission.^[^ ^18^ ^]^ Copyright 2016, IOP Publishing.

Among numerous aquatic organisms, jellyfish and starfish are the simplest structures, often chosen as the bioinspiration for inexpensive soft robots (**Figure** **2**a,b). Despite their simple structure and tissues of extremely low modulus, they can perform multi‐gait movements, such as catching objects and defending themselves by manipulating surrounding flows.^[^ ^11^ ^]^ Octopus is the most popular source of inspiration, since they are armed with astonishing abilities, such as, to undulate in tightly confined spaces, to adhere to uneven surfaces, to capture moving objects at distances, and to camouflage itself through changes in shapes, colors, and surface textures (Figure 2c). Manta rays and snailfishes can generate highly efficient thrusts by flapping their pectoral fins, and it becomes a long pursuit for making robotic fishes swimming amazingly fast (Figure 2d,e).^[^ ^12^, ^13^, ^14^ ^]^ Other locomotion modes, such as jetting inspired by squids, water‐walking inspired by striders (Figure 2), jumping inspired by frogs (Figure 2f),^[^ ^15^ ^]^ oscillating inspired by eel (Figure 2g),^[^ ^16^, ^17^ ^]^ are also sources of imitation in soft robotic design. When locomoting and maneuvering in water, avoiding obstacles is also important. Sea turtle combines both the flapping and twisting of flippers. The complex 3D motion of its flippers makes it the fastest and agilest reptile (Figure 2h).^[^ ^18^ ^]^

The aim of a soft robot is to replicate some of the motions and functionalities of soft‐bodied animals, namely, multi‐gait locomotion and manipulation. It is not the key to mimic the in vivo biological mechanisms by which these functions are achieved. For a simple aquatic organism, such as jellyfish, its structure is widely used for gripping various delicate and deformable objects; for fishes, swimming modes including flapping, undulating and jetting are imitated in different robotic fishes. Aside from basic actuations, moving, gripping, and additional functionalities such as sensing, camouflaging, and adapting to environmental changes are also key for achieving original aspirations of soft robots. These functionalities of current aquatic soft robotics will be discussed in the following sections.

Through multibillion years of evolution, marine species with soft bodies have developed ubiquitous strategies for underwater tasks, becoming inexhaustible fuels for current soft‐robotic research, and, in turn, man‐made robotic marine creatures may offer a new approach for exploring deep sea.

## 3 Soft Matter Materials and Design Strategies

Soft robots are composed of deformable soft matter materials, including elastomer, hydrogels, alloys, and fluids. These materials are lightweight, compliant, resilient, and easily deformable in 3D, with elastic, plastic, and rheological properties that could match those of natural organisms.

The first class of soft materials used for soft robots is silicone elastomers. Deformable elastomers are embedded with networks of perfusable chambers and channels, within which pressurized gases or liquids can pass through, causing inflation of chambers and channels.^[^ ^23^ ^]^ By attaching this layer of deformable microfluidic channel with a layer of less inflatable elastomer, the elastomer device exhibits unilateral bending upon pressurization. A typical combination of inflatable and less inflatable elastomers are Ecoflex and poly(dimethyl siloxane) (PDMS).^[^ ^9^ ^]^ Instead of choosing elastomers with different rigidities, the mismatching in rigidity can also be achieved by doping filler particles and fibers in elastomers. A pair of layers with mismatched rigidities generates a facile control over bending and thus actuation motion. The elaborate design of networks and segments of inflated microfluidic channels and chambers dictates the nature of the motion. This type of soft robots is also known as Pneumatic Networks actuators (PneuNets). Since silicone elastomers are water‐tight, they are indispensable seals for underwater soft robots.

To endow soft robots with adaptability, smart materials that respond to various stimuli are extensively adopted. These smart materials can expand or shrink upon thermal, electrical, light, and moisture/solvent triggers. For instance, thermo‐responsive and pH‐responsive hydrogels swell upon water retention and shrink upon water loss, triggered by temperature and pH changes, respectively; liquid crystalline elastomers exhibit large deformation under illumination of visible light.

Owing to the fast and pronounced response, the electrical trigger is the most commonly adopted stimulus. It needs to be induced via compliant electrodes. For instance, for a layer of ionic polymer between two flexible electrodes, by employing voltage, migration and redistribution of ions give rise to an osmotic pressure gradient, resulting in a unilateral bending of the whole structure. The same happens for a membrane of electroactive polymer containing electrolytes upon voltage excitation via electrodes. This is also referred to as the ionic‐polymer/metal composites (IPMCs) actuator. Similarly, when a dielectric elastomer (DE) is sandwiched between two flexible electrodes, an applied electric field causes a strong attraction between electrodes, squeezing the DE and leading to reduction in its thickness and expansion in its area. Actuators based on this mechanism is also known as DE actuator (DEA). If the DE is replaced by a dielectric liquid, which may endow with an actuator the self‐healing ability with immediate recovery of functionality after numerous dielectric breakdown events.^[^ ^24^, ^25^, ^26^ ^]^ Both IPMSCs and DEAs are frequently utilized in underwater soft robots.

Shape memory materials are a type of smart stimuli‐responsive materials that not only deform in three dimensions upon external triggers but also can spontaneously recover to their original shapes after excitations. For instance, shape memory alloy (SMA) undergoes reversible phase transitions upon each heating and cooling. They are often made into straight, twisted, coiled and bundled fibers to provide contraction, torsion, and bending motions. Both SMA and shape memory polymers are unutilized in wet environments.

**Figure 3:**
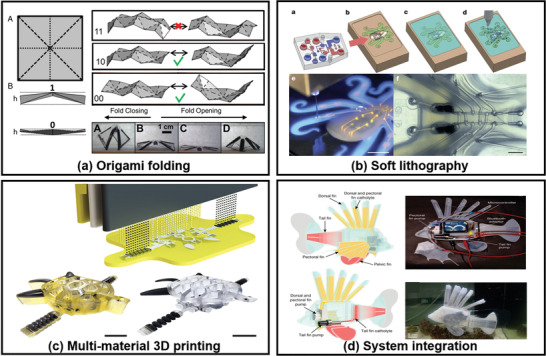
The examples of representative methods for fabricating soft robots: a) origami folding. Reproduced with permission.^[^ ^343^ ^]^ Copyright 2018, National Academy of Sciences. b) soft lithography. Reproduced with permission.^[^ ^344^ ^]^ Copyright 2016, Springer Nature. c) multi‐material 3D printing. Reproduced with permission.^[^ ^32^ ^]^ Copyright 2021, American Association for the Advancement of Science. d) system integration, Reproduced with permission.^[^ ^33^ ^]^ Copyright 2019, Springer Nature.

To facilitate multigait motion modes, rational design of 3D complex architectures with multiple materials of programmable strains is necessary. Inspired by nature, patterning different smart materials of distinctive mechanical properties on a single planar sheet can create complex 3D objects by origami folding.^[^ ^27^ ^]^ This “fabricating 2D and folding into 3D” approach facilitates fast and efficient prototyping and product iteration (**Figure** **3**a). SMA, shape memory polymers, and stimuli‐responsive polymers are often used for origami self‐folding. For example, a planar sheet of liquid crystal polymer with patterned crosslinked and un‐crosslinked domains can fold into an actuator. This actuator can be light‐fueled to walk and optically reconfigured to adopt different locomotion behaviors, such as changing moving directions.^[^ ^30^ ^]^ By selectively modifying an elastomer surface with active particles, the surface self‐folds into ferromagnetic origami robots with functions including swimming, multimode‐locomoting, and delivering objecting.^[^ ^30^ ^]^

Origami approach of “fabricating 2D and folding into 3D” is ingenious, but direct fabrication of 3D soft robots is versatile and compatible with various designs. Typical 3D fabrication techniques are “top down” soft lithography and “bottom up” 3D printing. Soft lithography is well established to process silicone elastomer into microfluidic chips with sophisticated designs, and it is widely used for fabricating composite fluid‐elastomer structures, such as PneuNets and grippers. Actuation is then realized by pressurzing fluids into networks of microchannels. For example, an octopus robot utilized gas generated from fuel catalytic decompositions to inflate the downstream channels for untethered actuation (Figure 3b).^[^ ^344^ ^]^ Not only pressurized fluids can flow through networks of microchannels for actuation, color‐changing fluids, conductive fluids, chemical combustive fluids, and stimuli‐responsive fluids can be also incorporated in microfluidic channels of soft robots to achieve camouflaging and locomotion. For instance, a color‐changing and walking soft robot is realized by two layers of microfluidic channel in a thin elastic Ecoflex. The soft robot walks when fluids are pumped into one layer, and it displays colors for disguise when thermo‐responsive pigment dispersions are perfused in another layer of microchannels.^[^ ^31^ ^]^

Most soft materials such as elastomers, electroactive polymers, and hydrogels are suitable for 3D printing.^[^ ^28^ ^]^ For instance, by using two photocurable silicone resins with different stretchabilities, PDMS‐based gripper actuated by compressed fluids has been fabricated via multi‐material digital light processing. Actuators can also be printed utilizing two types of shape memory polymers with different glass transition temperatures, or a shape memory polymer and an elastomer, via multi‐material stereolithography.^[^ ^29^ ^]^ Functional modules such as actuators, sensors, and power modules can be 3D printed, but a fully 3D‐printed soft robot is challenging. With further development of multi‐materials 3D printing, a fully integrated robotic turtle with modular components of actuator, circuitry, and interconnects is fabricated during a single print. Constant‐flow inputs can be converted to periodic oscillations of limbs (Figure 3c).

To fabricate a sophisticated soft robot capable of performing challenging tasks, techniques such as multi‐material 3D printing, shape deposition manufacturing, soft lithography, and even hard robotic techniques need to be adopted altogether. A complex robotic system includes actuation, sensing, power, and control modules. Each of these modules may be fabricated by different techniques and integrated together, analogous to hard robots. For instance, an electronic fish swimming in deep sea is composed of DE actuator for its flapping fins, polymer‐encapsulated power unit, voltage amplifier, and control units, all embedded in soft elastomer shells in a decentralized manner. To reduce weight, conventional rigid battery can be replaced by flexible redox flow battery. For instance, in a lionfish‐inspired aquatic robot, a flow‐cell battery supplies power to the pump and electronics, enabling the robotic fish to swim and fan its pectoral fins for communication (Figure 3d).

## 4 Biomimetic Functions and Potential Applications

Construction of simplified, lightweight, adaptive, and energy‐efficient soft robots is supposed to be a powerful tool for performing diverse tasks in wet environments. This section will start with the introduction of different actuation strategies with their corresponding disadvantages and advantages. The comparison of their performance will be also given. It aims at providing a guidance to choose a suitable actuation method for different scenarios. Actuation is fundamental to robots just like muscles to living creatures, through which the robot can perform the most basic deformations of contraction, expansion, or bending. Upon delicate designs and integrations, superior bionic tasks such as locomotion, gripping, sensing, and color display and camouflage can be realized. Recent studies on achieving these tasks will be reviewed subsequently. Particular attention will be given to those soft robots used in wet environments.

### 4.1 Actuation

The most fundamental function of robot is actuation, which is equivalent to the function of muscle that contracts, extends, and bends. A typical actuator, also known as artificial muscle, is the work horse of soft robots.^[^ ^34^ ^]^ It typically needs external stimuli, such as water,^[^ ^35^, ^36^, ^37^, ^38^, ^39^, ^40^, ^41^, ^42^, ^43^, ^44^, ^45^ ^]^ pH,^[^ ^46^, ^47^, ^48^, ^49^, ^50^ ^]^ heat,^[^ ^47^, ^51^, ^52^, ^53^, ^54^, ^55^, ^56^, ^57^ ^]^ light,^[^ ^58^, ^59^, ^60^, ^61^, ^62^, ^63^, ^64^, ^65^ ^]^ electricity,^[^ ^53^, ^66^, ^67^, ^68^, ^69^, ^70^, ^71^ ^]^ and magnetic field to perform.^[^ ^27^, ^72^, ^73^, ^74^, ^75^ ^]^ In recent years, soft actuators can be classified based on materials as elastomeric pneumatic actuator (PA), hydrogel actuator (HA), bio‐hybrid actuator (BHA), actuators made of DE, twisted and coiled yarns (TCY), SMA, liquid crystal elastomers (LCE), and ionic polymer‐metal composites (IPMC).^[^ ^76^ ^]^ Extensive efforts in design and fabrication are dedicated to improving the performance of artificial muscles, such as efficiency,^[^ ^42^, ^77^, ^78^ ^]^ power density,^[^ ^79^, ^80^, ^81^ ^]^ actuation force,^[^ ^82^, ^83^, ^84^, ^85^ ^]^ and the response speed^[^ ^66^, ^82^, ^86^, ^87^, ^88^, ^89^ ^]^ (**Table** **1**).

**Table 1:** Comparison of actuation method of soft robots. Bold denotes better performance. Adapted with permission.^[^ ^90^ ^]^ Copyright 2018, Springer Nature

Actuation method	Strain [%]	Work density [kJ m^−3^]	Modulus [MPa]	Power density [kW m^−3^]	Strain rate [% s^−1^]	Frequency [Hz]	Auxiliary equipment
Skeletal muscle	20–40	8–40	10–60	50–300	10–50	1–10	Body metabolism
PA	10–40	1–200	0.1–100	10–10^3^	10–70	1–5	Pneumatic pump, values
LCE	10–50	1–180^[^ ^55^ ^]^	0.1–3	0.01–10	1–10	0.001–1	Light, heat
BHA	10–25	0.1–10	**0.01–1**	1–10	10–100	1–5	Biocompatible medium
SMA	4–8	**10^4^–10^5^**	28–75 × 10^3^	**10^3^–10^5^**	10–50	0.5–5	Power supply
IPMC	0.5–10	1–10	25–2.5 × 10^3^	0.01–1	1–3	0.1–2	Power supply
DE	**1–10^3^**	10^2^–3.5 × 10^3[^ ^91^ ^]^	0.1–3	**10^3^–10^5^**	**10^2^–10^5^**	**1–100**	Power supply
HA	<87^[^ ^84^, ^92^ ^]^	10^−2[^ ^83^ ^]^–10^2[^ ^93^ ^]^	**10^−3^–0.1** ^[^ ^94^ ^]^	10^−5[^ ^95^ ^]^–35^[^ ^96^ ^]^	–	<1^[^ ^97^ ^]^	Light, heat, pH, water, etc.
TCY	0.2–90^[^ ^98^, ^99^, ^100^, ^101^, ^102^, ^103^ ^]^	**2.5 × 10^3^** ^[^ ^104^ ^]^ **–1.6 × 10^4^** ^[^ ^105^ ^]^	2.4 × 10^3[^ ^91^ ^]^	1.3 × 10^3[^ ^105^ ^]−^5 × 10^3[^ ^104^ ^]^	15^[^ ^98^ ^]^–50^[^ ^103^ ^]^	2–12^[^ ^79^, ^99^, ^100^, ^106^ ^]^	Heat, solvent

**Figure 4:**
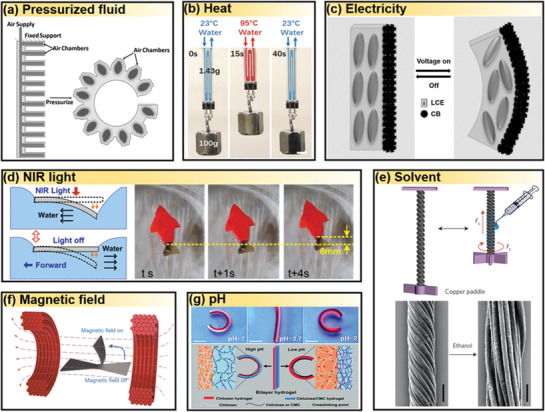
Actuation methods by external stimuli through: a) pressurized fluid. Reproduced with permission.^[^ ^132^ ^]^ Copyright 2021, Springer Nature. b) heat. Reproduced with permission.^[^ ^133^ ^]^ Copyright 2018, Wiley‐VCH. c) electricity. Reproduced with permission.^[^ ^345^ ^]^ Copyright 2020, American Chemical Society. d) light. Reproduced with permission.^[^ ^135^ ^]^ Copyright 2018, American Chemical Society. e) solvent. Reproduced with permission.^[^ ^136^ ^]^ Copyright 2015, Springer Nature. f) magnetic field. Reproduced with permission.^[^ ^137^ ^]^ Copyright 2020, Springer Nature. g) pH. Reproduced with permission.^[^ ^46^ ^]^ Copyright 2017, Royal Society of Chemistry.

PA are devices in which pressurized fluids expand deformable chambers and channels, converting the energy of compressed fluids into mechanical actuation of objects (**Figure** **4**a).^[^ ^107^, ^108^ ^]^ PA can generate a power density as high as 10^3^ KW m^−3^ and an actuation force up to 1 MPa,^[^ ^91^ ^]^ and it can be used in extreme conditions, such as deep sea. The major limitation of PA is its bulky appearance, largely owing to the size of the fluid tank,^[^ ^109^, ^110^ ^]^ and the actuation is generally slow and inefficient.^[^ ^111^ ^]^ Additionally, the nonlinear relationship between pressure and contraction ratio, as well as the hysteresis characteristics of PA may lead to a low accuracy of modeling and control.^[^ ^112^ ^]^

Actuation can also be achieved by stimulus‐responsive soft materials, which change their sizes and shapes, reversibly via swelling/deswelling, upon various external stimuli in ambient environment. SMA, LCE, and TCYs can be actuated by temperature changes (Figure 4b). Reversible phase transitions of SMA and LCE during heating and cooling cycles result in actuation, thus, the heat storage and release in these materials typically slow their actuation speed and the response frequency.^[^ ^90^ ^]^ TCY actuators refer to coiled yarns of nylon, carbon, and fishing lines, which are thermally activated by hot fluids, joule heat produced by electrical current or heating wire. Upon heating, the volumetric expansion of TCY results in contraction or bending. TCY actuators are capable of generating high output stress, power, and work densities with long working life. The thermal actuation typically has low energy conversion efficiency and thus consumes remarkable amount of energy. Also, it usually takes a long time for heat transfer, the response speed and frequency for this type of actuation is low.

The fastest responsive actuators are electrically triggered ones. IPMC and DEs are representative examples that can be actuated by applying an electric field (Figure 4c). DE actuators are featured by high power density, fast response speed, large strain and strain rate, good reliability, and long lifetime.^[^ ^113^, ^114^, ^115^, ^116^, ^117^, ^118^ ^]^ Underwater, the weight of the DE actuator is balanced by buoyance and its surrounding water can be used as an electric ground, which enables high payload^[^ ^119^ ^]^ and even untethered swimming of soft robots.^[^ ^120^ ^]^ One concern of DE actuators is its high operating voltage (typically thousands of volts), which can cause safety problem for biomedical and wearable applications.^[^ ^91^ ^]^

In contrast to DE, IPMC actuators only require a low operating voltage of 1–5 voltages.^[^ ^121^, ^122^ ^]^ The three‐orders‐of magnitude reduction in actuating voltage lies in their compositions. The responsive material sandwiched by two electrodes is composed of ionic polymer and electrolyte for IPMC, while in DE it is composed of a DE. In IPMC, only a few voltages can drive the electrolysis of electrolyte solution, leading to ion migration, generating osmotic pressure gradient, and making IPMC bending. It is naturally suitable for IPMC actuators to be used in wet environments due to the presence of aqueous electrolyte.^[^ ^123^ ^]^ IPMC is frequently used as a caudal fin for propulsion in underwater soft robots.^[^ ^124^, ^125^, ^126^ ^]^ However, the flapping frequency of IPMC fins is restrained by the speed of ion migration, which in turn limits the propulsion speed of the soft robot.^[^ ^127^ ^]^

Hydrogel‐based actuators are a class of actuators that swell by gaining water and shrink by losing water, upon various stimuli, including water, light, pH, and biomolecules (Figure 4d–g). Hydrogel has a near 90% of water content, and its softness makes them suitable for constructing underwater and in vivo robots.^[^ ^90^ ^]^ For instance, it is extensively used for creating nondestructively gripping robots for fragile and delicate objects, since HA may minimize the risk of damaging target objects.^[^ ^128^ ^]^ In addition, HA is highly designable and programmable to swell and deswell in response to an external stimulus.^[^ ^129^, ^130^ ^]^ The major challenge for HA is that its current energy density, power density, and endurance are far lower than those of biological muscles.^[^ ^131^ ^]^

### 4.2 Locomotion and Swimming in Aquatic Environments

**Figure 5:**
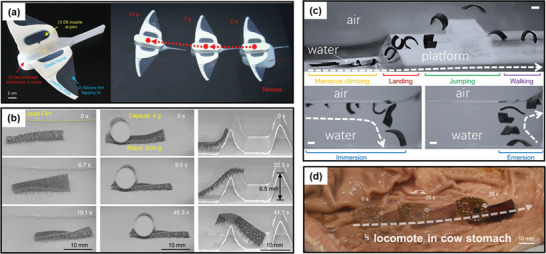
Illustrations of some soft robots locomoting in wet environments: a) a soft robotic fish using DEs as flapping fins that swims freely in the Mariana Trench at a depth of 10900 m and in the South China Sea at a depth of 3224 m. Reproduced with permission.^[^ ^172^ ^]^ Copyright 2021, Springer Nature. b) a soft robot with multiple hydrophobic tapered feet that moves on wet surface with liquid film. Reproduced with permission.^[^ ^346^ ^]^ Copyright 2018, Springer Nature. c) a small‐scale soft robot exhibiting a multimode locomoting ability in water and on land. Reproduced with permission.^[^ ^188^ ^]^ Copyright 2018, Springer Nature. d) a multi‐legged soft robot moving in a wet cow stomach. Reproduced with permission.^[^ ^191^ ^]^ Copyright 2020, Wiley‐VCH.

Soft robots that can autonomously move in aqueous environments have been developed for a spectrum of purposes, such as exploring sea and seabed conditions, investigating marine life, detecting ocean intruders, and recording ocean currents (see examples in **Figure** **5**).^[^ ^119^ ^]^ Soft robots operate in unsafe and unpredictable aqueous environments, thus they ought to cope with risk of mechanical failure under dynamic conditions and complexity of navigation in unstructured submerged scenarios.^[^ ^138^, ^139^ ^]^ Locomotion of soft robots can be achieved either by rigid motors or stimuli‐responsive soft actuators.^[^ ^7^ ^]^ Rigid motors are effective, but the rigidity impedes a lightweight and flexible integration,^[^ ^120^ ^]^ which are not enclosed in the current review. Interested readers may refer to other reviews for more details.^[^ ^140^, ^141^ ^]^

In aqueous environments, hydrogels can take up water and swell by external stimulus. The reversible swelling‐deswelling property of hydrogels by stimuli can be exploited to produce macroscopic actuation for soft robots. A large variety of stimuli, such as heat,^[^ ^142^, ^143^, ^144^, ^145^, ^146^, ^147^, ^148^, ^149^ ^]^ light,^[^ ^150^, ^151^, ^152^, ^153^ ^]^ water,^[^ ^154^, ^155^ ^]^ pH,^[^ ^156^, ^157^, ^158^ ^]^ biomolecules,^[^ ^159^, ^160^ ^]^ and electric field,^[^ ^157^, ^161^, ^162^, ^163^ ^]^ can change the osmotic pressure of solvents in hydrogels and/or environments, resulting in water diffusion in or out of the hydrogels, corresponding to their expansion or contraction, respectively.^[^ ^164^ ^]^ Other than common physical stimuli, a recent study showed that a hydrogel robot could locomote on water surface for 3.5 h by the Marangoni effect (Figure 5e).^[^ ^165^ ^]^ This effect has been widely used for self‐propelling robots, where they locomote by an interfacial flow from low surface tension domain toward high surface tension domain.

Besides hydrogels, other soft stimuli‐responsive materials and pneumatic devices^[^ ^108^, ^166^, ^167^, ^168^ ^]^ have been also used for locomoting soft robots, including DEs,^[^ ^119^, ^120^, ^134^, ^169^, ^170^, ^171^, ^172^, ^173^, ^174^ ^]^ ionic polymer metal composites (IPMCs),^[^ ^124^, ^125^, ^126^, ^175^, ^176^, ^177^, ^178^, ^179^ ^]^ and SMAs.^[^ ^180^, ^181^, ^182^ ^]^ These robots can reach the speeds of 0.35 body length per second (BL s^−1^) by SMAs,^[^ ^183^ ^]^ 0.25 BL s^−1^ by IPMCs,^[^ ^176^ ^]^ 0.69 BL s^−1^ by DEs,^[^ ^120^ ^]^ and 0.78 BL s^−1^ by PAs^[^ ^184^ ^]^ in aqueous environments.

Despite recent advances, it still remains challenging to achieve fast locomotion speed. Compared with the moving speed of animals and rigid robots, 1 to 100 BL s^−1^, the reported locomotion speed of soft robots in literature is less than 0.8 BL s^−1^.^[^ ^184^ ^]^ This is partially owing to intrinsic nature of soft materials constituting the robots.^[^ ^185^ ^]^ Mechanical energy transfers slower in soft materials than hard materials, resulting in a slower locomotion of soft robots.^[^ ^186^ ^]^ Moreover, soft materials typically exhibit nonlinear mechanical properties. For instance, their elasticity can change remarkably upon a threshold strain, adding difficulty to accurately model and control the locomotion of soft robots.

### 4.3 Gripping

Biological sampling of ocean species is important for studying sea ecosystem, biological diversity, and genetic adaptions. However, collecting intact samples of delicate sea creatures has been a challenge, since existing technologies, such as nets and vacuum devices, often damage their integrity during capture.^[^ ^192^ ^]^ Soft gripping robots provide a safer way for this task. A soft gripper should be sufficiently compliant to avoid any damage to fragile objects, and it also should conform to objects with complex shape to handle them reliably.

**Figure 6:**
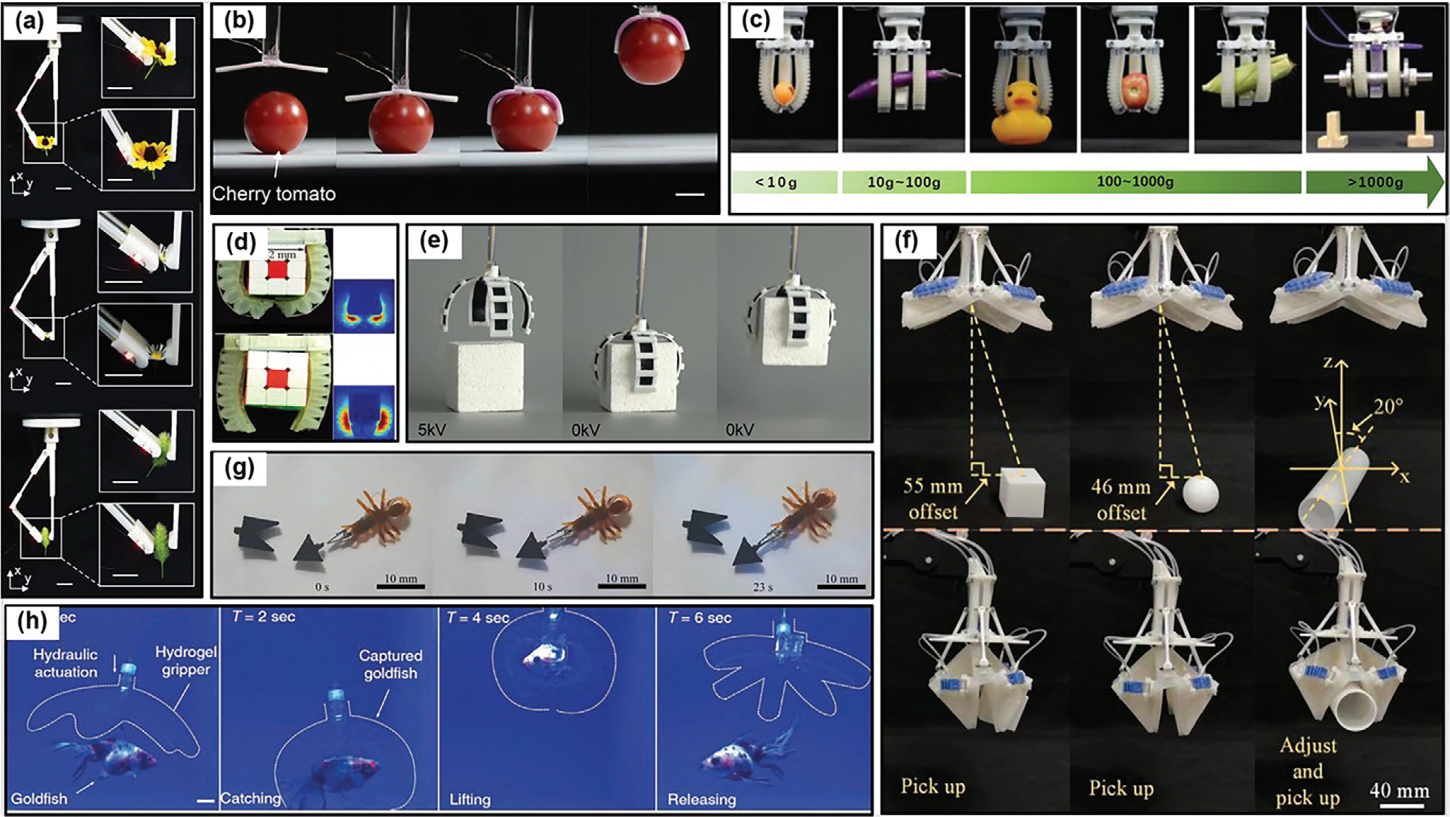
Demonstrations of soft grippers consisting of different materials: a) a SMA gripper to pick up two daisy‐like flowers with different sizesand a panicle of green bristlegrass. Reproduced with permission.^[^ ^352^ ^]^ Copyright 2021, Elsevier. b) a LCE gripper to grip and lift a cherry tomato. Reproduced with permission.^[^ ^353^ ^]^ Copyright 2021, American Chemical Society. c) an SMP gripper capable of grasping and lifting a wide range of object weights from 10 to 1500 g. Reproduced with permission.^[^ ^243^ ^]^ Copyright 2019, Wiley‐VCH. d) a soft gripper collecting Rubik’s cube by a pressurized air input. Reproduced with permission.^[^ ^354^ ^]^ Copyright 2020, IOP Publishing. e) a DE gripper approaches the cube and then grips it by turning off the voltage. Reproduced with permission.^[^ ^355^ ^]^ Copyright 2019, Elsevier. f) a soft pneumatic gripper to grasp a cube, sphere and cylinder. Reproduced with permission.^[^ ^357^ ^]^ Copyright 2021, IOP Publishing. g) IPMC gripper robots splice components cooperatively. Reproduced with permission.^[^ ^356^ ^]^ Copyright 2019, IOP Publishing. h) a transparent hydrogel gripper to catch, raise and release a live goldfish. Reproduced with permission.^[^ ^128^ ^]^ Copyright 2017, Springer Nature.

Similar to human using fingers to hold an object, soft robots grip by bending themselves around the object. There are two types of grippers: one is a soft actuator that can autonomously bend and cap the target object upon a stimulus, which is often achieved by stimuli‐responsive soft materials; the other is a soft gripper that cannot actuate autonomously and hence is powered by an external actuator. The maximum mass of an object that the gripper can elevate and the minimum time it takes to complete the task are two basic criteria for evaluating the performance of grippers, which are given in **Table** **2** with respect to their constituting materials (**Figure** **6**a–h).

**Table 2:** Comparison of soft grippers of their performance (FE: fluidic elastomer; DE: dielectric elastomer; IPMC: ionic polymer‐metal composites; SMA: shape memory alloy; PG: pneumatic gripper; SMP: shape memory polymer; LCE: liquid crystal elastomer)

Category (By materials)	Lifting ratio (Object mass/gripper mass)	Gripper size [10^−2^ m]	Response time [s]
FE	2^[^ ^193^ ^]^–68^[^ ^194^ ^]^	0.5^[^ ^195^ ^]^–120^[^ ^196^ ^]^	0.1^[^ ^197^ ^]^
DE	5.5^[^ ^198^ ^]^–100.8^[^ ^199^ ^]^	2^[^ ^200^ ^]^–10.3^[^ ^201^ ^]^	0.1^[^ ^201^ ^]^
IPMC	2^[^ ^202^ ^]^–3.5^[^ ^203^ ^]^	0.5^[^ ^203^ ^]^–8^[^ ^204^ ^]^	0.33^[^ ^205^ ^]^
SMA	15^[^ ^206^ ^]^–25.8^[^ ^207^ ^]^	0.9^[^ ^208^ ^]^–11.5^[^ ^209^ ^]^	0.15^[^ ^206^ ^]^
PG	13^[^ ^210^ ^]^–38.3^[^ ^211^ ^]^	0.5^[^ ^195^, ^212^ ^]^–148^[^ ^213^ ^]^	0.1^[^ ^197^ ^]^
SMP	48.9^[^ ^214^ ^]^	0.4^[^ ^215^ ^]^–6^[^ ^216^ ^]^	10^[^ ^217^ ^]^
LCE	210^[^ ^218^ ^]^	0.03^[^ ^219^ ^]^–3^[^ ^220^ ^]^	0.2^[^ ^221^ ^]^

Gripping an object that is ultra‐smooth, ultra‐thin, or highly greasy is extraordinarily difficult. In these situations, special strategies should be adopted when designing the gripper. Inspired by octopus, which uses tentacles covered with suction cups to grasp objects underwater, electro‐adhesion is a useful method. It exploits the attraction force between the object and integrated electrode in gripper.^[^ ^199^, ^222^, ^223^, ^224^, ^225^, ^226^, ^227^, ^228^ ^]^ It generally requires a high voltage, on the order of a few kilovolts, and can produce high holding force. For instance, the generated normal and shear stresses are in the range of 4–13 and 12–62 kPa,^[^ ^227^, ^229^, ^230^, ^231^ ^]^ respectively. By optimizing the geometries of the electrodes and the insulating layer, the performance of electro‐adhesion can be further improved.^[^ ^229^, ^232^, ^233^ ^]^

Soft gripper typically exhibits small grasping force.^[^ ^234^, ^235^, ^236^ ^]^ To lift heavy objects, soft materials with variable stiffness, including low‐melting point alloys,^[^ ^198^, ^237^, ^238^, ^239^, ^240^, ^241^ ^]^ shape memory materials,^[^ ^214^, ^242^, ^243^, ^244^ ^]^ electrorheological and magnetorheological fluids^[^ ^245^ ^]^ are adopted. Generally, the gripper is soft when approaching the object, after enveloping the target, it suddenly stiffens and holds the target by caging. A stiffness‐tunable soft gripper of shape memory materials is fabricated and it finishes its softening‐stiffening cycle within 32 s.^[^ ^243^ ^]^ The gripper could enhance its stiffness by 120 times and pick up the objects of the weights ranging from 10 g to 1.5 kg (Figure 6f).^[^ ^243^ ^]^

Most existing soft grippers are challenging when operating under high‐pressure, extreme temperatures, and corrosive environments.^[^ ^213^ ^]^ A soft gripper operates in deep sea at depths over 1200 m with ambient pressure exceeds 100 atmospheres is developed, where it can produce a pulling force larger than 35 N on an object.^[^ ^246^ ^]^ However, the effects of position, geometry, and support of the sample on the performance of the gripper remain to be addressed.

### 4.4 Sensing

Sensing empowers soft robots with capability to continuously detect and regulate their motions, for adaptations, autonomy, and interactions with humans and environment.^[^ ^4^, ^5^, ^6^ ^]^ Sensing is exceptionally important for interactive soft robots operating in wet, dynamic, and complex environments.^[^ ^248^ ^]^ Sensors mounted on rigid bodies cannot be used directly on soft robots, due to the mechanical mismatch between sensors and soft robots. Fortunately, flexible sensors have been rapidly developed in recent years, which are composed of a variety of sensitive materials, such as metals,^[^ ^249^, ^250^, ^251^ ^]^ carbons,^[^ ^252^, ^253^, ^254^, ^255^, ^256^, ^257^ ^]^ oxides,^[^ ^258^, ^259^, ^260^ ^]^ and stimuli‐responsive gels.^[^ ^261^, ^262^, ^263^, ^264^ ^]^ These flexible sensors can be integrated with underwater soft robots, from which a variety of information ranging from physical, chemical, and biological signals can be perceived. For example, a soft somatosensitive HA that could perform feedback‐controlled gripping is reported.^[^ ^265^ ^]^ When the actuator perceived and gripped three objects of different sizes under NIR illumination, both the time and magnitude of resistance change by piezoresistive effect were distinct for different sized objects, providing the potential for shape recognition of unknown objects.^[^ ^265^ ^]^ A closed‐loop control system to regulate shrinking and relaxation motions of the octopus‐like arm was also developed.^[^ ^265^ ^]^

To precisely control and locate a robot in aquatic environments, whisker‐inspired sensor arrays have been developed.^[^ ^266^, ^267^, ^268^, ^269^ ^]^ These sensors are dense arrays which are distributed over the surface of the robot. In this way, the robot can perceive relevant information of interest precisely and make a response. For example, sea anemones are able to avoid being broken or swept away by their surrounding flowing seawater.^[^ ^270^ ^]^ The array of tentacles of anemones can sensor the flow velocity and inform its body to shrink to protect itself at a fast water flow. Inspired by the sea anemone, a NdFeB/Ecoflex composite robot combining shape‐deforming and sensing capabilities was developed. The top tentacle‐like magnetoelectric sensors could perceive surrounding flow velocity, and the bottom magneto‐stimulated shrinkable body can deform accordingly.^[^ ^270^ ^]^

Recent advances in electronic skins have significantly promoted sensing capability and diversity of soft robots.^[^ ^3^, ^271^, ^272^, ^273^, ^274^, ^275^, ^276^, ^277^, ^278^, ^279^ ^]^ This is mostly achieved by capacitive or resistive strain sensors,^[^ ^280^, ^281^, ^282^, ^283^ ^]^ or optical devices like optical fibers and cameras to detect deformations.^[^ ^284^, ^285^, ^286^ ^]^ However, sophistication of current skin sensors may not be comparable to that of human skin receptors.^[^ ^3^ ^]^ Considering interactive robots in wet environments may experience complex deformations, large amounts of information during tasks in unknown or dynamic environments need to be collected and processed, such as abyssal exploration and sampling, more sophisticated sensors are required to be developed. Additionally, it is also necessary to extract useful information from vast amount of data collected by sensors. Machine learning could play an important role in bringing sensing to human‐like performance levels.^[^ ^3^, ^287^ ^]^

### 4.5 Color Display and Camouflage

To closely investigate marine life within their habitats with minimal disturbance for long‐term, camouflage soft robot that can locomote alongside with marine life without alarming them is an ideal tool. A diversity of living creatures can change their color and morphology, which endows them with the adaptive abilities for camouflage, display, or communication.^[^ ^288^, ^289^, ^290^, ^291^, ^292^ ^]^ For example, sea animals like jellyfish and leptocephalus can detect external stimuli and camouflage themselves to escape from predators.^[^ ^293^, ^294^, ^295^ ^]^ Inspired by these sea animals, various soft robots with the capability of color display and camouflage have been developed and will be reviewed in this section.

#### 4.5.1 Color Display

**Figure 7:**
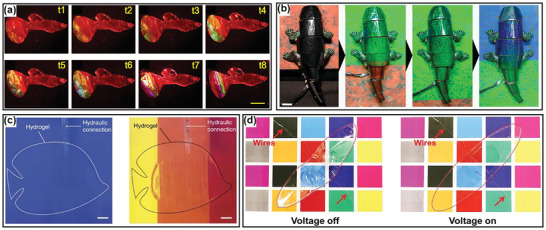
Examples of soft robots with the capability of color display and camouflage: a) a soft robotic fish actuated by cardiomyocyte beating with the tail showing variable structural color during one myocardial cycle. Reproduced with permission.^[^ ^299^ ^]^ Copyright 2019, American Chemical Society. b) a biomimetic chameleon soft robot that can detect and match the local background color in real‐time. Reproduced with permission.^[^ ^323^ ^]^ Copyright 2021, Springer Nature. c) a transparent hydrogel robotic fish to camouflage when moving over rainbow‐colored background. Reproduced with permission.^[^ ^128^ ^]^ Copyright 2017, Springer Nature. d) a soft transparent DEA robot camouflaging under the application of voltage. Reproduced with permission.^[^ ^324^ ^]^ Copyright 2019, Wiley‐VCH.

Nanostructured photonic materials can tune electromagnetic waves and control propagation of photons with the energy in their photonic band gap (PBG), resulting in brilliant structural colors.^[^ ^296^ ^]^ If the PBG is changed by an external stimulus in environment, the output color will be also changed, which is used as an effective strategy in soft robotic systems to display.^[^ ^296^, ^297^, ^298^, ^299^, ^300^, ^301^, ^302^, ^303^, ^304^ ^]^ The stimulus can be mechanical,^[^ ^305^, ^306^, ^307^ ^]^ chemical,^[^ ^308^ ^]^ electrical,^[^ ^309^, ^310^ ^]^ magnetic,^[^ ^311^ ^]^ light,^[^ ^312^ ^]^ or thermal.^[^ ^313^, ^314^ ^]^ The response time of color switching can be as fast as 0.1 millisecond to mechanical stimuli.^[^ ^307^ ^]^ Color displaying capacity can also be achieved by autonomic regulation.^[^ ^296^, ^299^, ^300^, ^315^ ^]^ For example, cardiomyocyte cells with repeated contraction were integrated into an intelligent soft robotic “heart‐on‐a‐chip”, which could visualize therapeutic effects of screened drug (**Figure** **7**a).^[^ ^296^, ^300^, ^315^ ^]^

Other than structural colors, dyes, pigments, and luminescent color‐switchable polymers can be employed.^[^ ^31^, ^49^, ^316^, ^317^, ^318^, ^319^, ^320^, ^321^, ^322^ ^]^ Directly embedding light‐emitting elements to mimic chromatophores in living species is more widely employed in soft robotic systems.^[^ ^4^ ^]^ Chromomorphism in artificial systems has been successfully replicated either by controlling pigment‐containing liquids in an artificial dermal melanophore, or using areal expansion of DE structures.^[^ ^322^ ^]^ By designing microfluidic networks which were then integrated on soft substrates, coloration was accomplished by hydrostatic translocation of pigmented fluids with different colors in microchannels.^[^ ^31^, ^317^ ^]^ Doping fluorescent substance into substrate materials endows soft robots with a variety of coloring patterns.^[^ ^49^, ^319^, ^320^ ^]^ Thin rubber sheets consisting of a ZnS phosphor‐doped DE layer sandwiched by layers of hydrogel electrodes were created.^[^ ^320^ ^]^ These sheets were able to vary illuminance under deformation and were integrated onto the skin of a soft robot, equipped with the functions of sensory feedback from outer surrounding and dynamic coloration.^[^ ^320^ ^]^

#### 4.5.2 Camouflage

The most simple strategy for camouflage is transparent soft robot (Figure 7b‐d).^[^ ^2^, ^120^, ^128^, ^324^, ^325^, ^326^ ^]^ If a soft robot is composed of entirely transparent components absorbing very little light, it can disguise itself in water as its refractive index is close to that of water (≈1.33).^[^ ^128^, ^324^ ^]^ DE actuators (DEAs),^[^ ^134^, ^324^, ^327^ ^]^ electrothermal actuators (ETAs),^[^ ^328^, ^329^, ^330^ ^]^ and stimuli‐responsive polymers and hydrogels^[^ ^128^, ^331^ ^]^ show a high transmittance across visible spectrum larger than 80%, thus they can be used for transparent actuators.^[^ ^2^ ^]^ For example, a DEA was invented by using an acrylic adhesive as the dielectric layer, an internal chamber filled with ionic fluid as one of the electrodes and surrounding liquid as another electrode, which has a transparency as high as 94%, similar to a leptocephalus.^[^ ^134^ ^]^

An active camouflage strategy refers to a soft robot changing its color actively adaptive to environment. This kind of soft robot needs to acquire prior information of background colors in its surrounding environment to match with them. The group of Whitesides demonstrated a pneumatically driven camouflaged soft robot in water.^[^ ^31^ ^]^ Camouflage was realized by pumping colored liquids through a network of microfluidic channels to match the background colors.^[^ ^31^ ^]^ However, this color matching was conducted manually by the operator, instead of an automatic system. To develop an intelligent robotic system, it should automatically sense surrounding colors and complete color‐shifting to adapt to the surrounding environment. For example, a highly integrated system can consist of three units, embedded color sensors to record color patterns of the surrounding environment, a smart program to analyze the acquired information from the sensors, and a feedback control system to guide the change of the appearance of robot accordingly.^[^ ^310^ ^]^

## 5 Challenges and Perspectives

Soft robotics emerges from a bioinspired idea to make robots soft, flexible, compliant, reconfigurable, and adaptable, like soft‐bodied biological organisms, while hard robots are heavy, rigid, and sophisticated machines. The application scenario for soft robots is distinctively different from hard robots. Soft robots are supposed to be more fitting for interacting with humans, handling fragile objects, and operating in dynamic environments.^[^ ^246^ ^]^ A soft robot is not driven by rigid motors, but like biological organisms, which is driven by artificial muscles. Moreover, together with the rapid development of skin electronics, seamless integration of soft tactile sensors and transducers in a soft robot, it will enable multiple sensing modalities that mimic biological systems, bringing us closer to a future of artificial‐organism‐like soft robots. Despite the great perspective and recent advances of soft robotics, there still need considerable endeavors ahead, for instance, intelligent material, novel design methodology, and bi‐directional control.

Intelligent materials that can switch between soft/compliant and stiff/load‐bearing states are demanded. This class of materials can provide either stability and high output or compliance and versatility as it needs.^[^ ^214^, ^243^, ^332^, ^333^ ^]^ Representative intelligent material is self‐regulating biological muscles. Inspired by this, current artificial muscle can progressively stiffen through mechanical training.^[^ ^84^, ^334^, ^335^ ^]^ In addition, self‐healing artificial muscle is also developed for coping with unpredictable environments.^[^ ^336^, ^337^, ^338^, ^339^, ^340^, ^341^, ^342^ ^]^

Structural design optimized by computer modeling is critical for embedding the functionality of soft robots. For instance, 3D‐printed pneumatic soft robot has demonstrated how shapes and configurations of air chambers can be manipulated to realize multigait behaviors. Morphological computation that emulates high‐order nonlinear systems is essential to find topology optimization approach for discovering origami fold patterns that result in optimal out‐of‐plane motion. Design optimization is also important for achieving a high‐speed swimming, trajectory planning, and precise grasping for soft robot. New design and modeling methodology are the key to robots with embodied mechanical intelligence.

System integration is often not emphasized sufficiently, but it is indispensable for lightweight, flexible, compact, and agile soft robots. The actuator, sensor, controller, and power components of a soft robot have distinct stiffnesses, to interface them efficiently and seamlessly becomes increasingly important, as the complexity of the soft robotic system increases.^[^ ^90^ ^]^ At system level, tactile sensing capability and bi‐directional control are the core for realizing organism‐like soft robot. Currently, most soft robots lack autonomy, closed‐loop, and bi‐directional control. In biological systems, living creatures can detect dangers through receptors to receive information, either light, sound, pain, or chemicals, which help them escape from predators and thrive in the world. To mimic these creatures, multimodal sensors that can provide feedbacks from surroundings are necessary. Achieving precise control after sensing and proprioception is also challenging, since the soft‐bodied robot has almost infinite degrees of freedom.^[^ ^6^ ^]^ Currently, AI and machine learning have been introduced to learn and predict the behavior of soft robot, in purpose of aiding in sensing and adaptive control of these systems. We envision that with synergistic developments of soft materials, novel soft robotic algorithms, and hardware, intelligent robotic systems with high level of planning, control, state estimation, and decision‐making can be possible.

## Conflict of Interest

The authors declare no conflict of interest.

## Author Contributions

Y.F.Z. and J.L.F. contributed equally to this work. C.Q. and J.H.X. conceived, designed, and supervised the review. Z.L. provides important insights for this review. C.Q. and T.T.K. wrote the manuscript. Y.F.Z., J.L.F., and K.L.L. conducted investigation and collected figures. All authors commented on the manuscript.
